# Effectiveness and Safety of Infliximab in Two Cases of Severe Chondrocalcinosis: Nine Years of Follow-Up

**DOI:** 10.1155/2014/536856

**Published:** 2014-11-11

**Authors:** Jácome Bruges-Armas, Bruno F. Bettencourt, Ana R. Couto, Manuela Lima, Ana M. Rodrigues, Nathan Vastesaeger, Matthew A. Brown

**Affiliations:** ^1^SEEBMO, Hospital de Santo Espírito da Ilha Terceira, 9700 Angra do Heroísmo, Portugal; ^2^Genetics & Arthritis Research Group (GARG), Institute for Molecular and Cell Biology (IBMC), 4150 Oporto, Portugal; ^3^Grupo de Epidemiologia e Genética Humana do Departamento de Biologia da Universidade dos Açores, 9500 Ponta Delgada, Portugal; ^4^Rheumatology Research Unit, Molecular Medicine Institute, Lisbon, Portugal; ^5^Serviço de Medicina Interna, Hospital de Santo Espírito da Ilha Terceira, 9700 Angra do Heroísmo, Portugal; ^6^Merck Sharp & Dohme, 1135 Brussels, Belgium; ^7^University of Queensland, Diamantina Institute, Translational Research Institute, Princess Alexandra Hospital, Brisbane, QLD 4192, Australia

## Abstract

*Objectives*. To investigate the efficacy of infliximab in the treatment of severe calcium pyrophosphate deposition diseases (CPPD). *Methods*. Two patients with severe CPPD and diffuse idiopathic skeletal hyperostosis- (DISH-) like phenotype are described. Both patients were resistant to therapy with nonsteroidal anti-inflammatory drugs (NSAIDs). Both patients were treated with infliximab, a TNF-*α* receptor antagonist, for nine years. *Results*. Treatment with infliximab resulted in major clinical and laboratory improvements without relevant side effects. *Conclusions*. These results suggest that infliximab may be an effective treatment of severe CPDD.

## 1. Introduction

Calcium pyrophosphate dehydrate deposition disease (CPPD) is caused by calcium pyrophosphate dehydrate (CPP crystals) crystal deposition. Three main forms of CPPD are usually described: acute pseudogout, inflammatory chronic arthropathy, and osteoarthritis-like disease [1]. An additional CPPD phenotype characterized by peripheral and axial enthesopathic calcifications was described by Resnick and others [2–5]. Pseudogout, characterized by acute attacks of inflammatory arthritis, mainly in wrists and knees, is sometimes severe and can be refractory to current nonbiological treatments. Treatment of CPPD depends on the clinical manifestations and disease severity, and several drugs may be used, like nonsteroidal anti-inflammatory drugs (NSAIDs), intra-articular or systemic steroids, methotrexate, hydroxychloroquine, and colchicine [6]. However, severe cases of CPPD refractory to classic treatments have been reported and, more recently, other therapeutic approaches have been described that may be effective in these conditions.

The administration of the recombinant IL-1R antagonist anakinra and other IL-1 targeting drugs has been shown to be effective in microcrystalline diseases [7]. The administration of anakinra (interleukin-1 receptor antagonist) or canakinumab (anti-interleukin-1 beta) to patients with acute gout led to rapid resolution of inflammation [8, 9] and anakinra treatment was efficacious in the treatment of two patients with severe episodes of pseudogout, preventing the occurrence of new flares of the disease if administered continuously in a patient with end-stage renal failure [10, 11].

Infliximab is a TNF-*α* receptor antagonist used successfully to treat severe forms of inflammatory arthritis, namely, ankylosing spondylitis [12] and rheumatoid arthritis [13]. Assuming that there is a role for TNF-*α* in crystal induced arthritis, we have treated with infliximab two cases of severe CPDD resistant to NSAIDs treatment.

## 2. Methods and Results

### 2.1. Case Report 1

A 30-year-old male patient presented with bilateral ankle swelling and pain. On examination, there was reduced range of motion (ROM) in both ankles causing gait difficulties and requiring adapted footwear. X-rays revealed narrowing of the articular space, sclerosis as well as bridging in both ankles, calcification of the iliolumbar ligament, and pelvic and femoral entheseal ossification.

**Figure 1:**
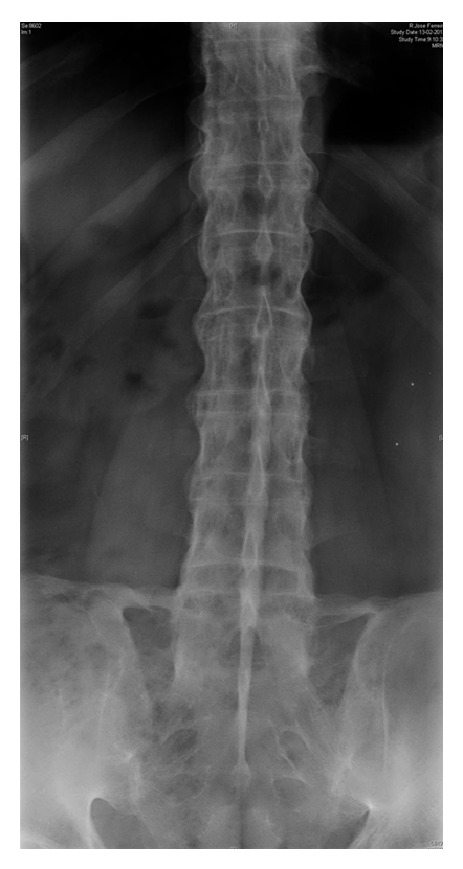
Lumbar spine and iliolumbar ligament calcification.

Subsequently, his arthritis followed a relapsing-remitting course, with several episodes of acute synovitis in knees, wrists, metacarpophalangeal joints (MCPs), shoulders, and spine. A diagnosis of CPDD/DISH was made at age 49 based on X-rays that showed huge anterior longitudinal ligament calcification of the entire spine and massive iliolumbar calcification with restriction of lumbar spine mobility (Figure 1). He also had bilateral gonarthrosis, patellofemoral arthrosis, fusion of both ankles, and huge femoral neck osteophytosis. He had a history of severe alcohol abuse. Laboratory tests identified high ferritin (>800 ng/mL) and erythrocyte sedimentation rate (ESR) of 60 mm/hr. Plasma and urine levels, fluoride, calcium, phosphate, and magnesium were normal. Moreover, serum levels of growth hormone (GH), parathormone (PTH), thyroid hormones (THs), and vitamin D were also normal. Rheumatoid factor and antinuclear antibodies (ANA) were normal or negative. Due to the high ferritin level,* HFE* mutations were investigated; he was a compound heterozygote for C282Y and H63D, but there were no excess liver iron stores in MRI. HLA-B27 was negative.* ANKH* sequencing found no pathogenic variants. There was no psoriasis, inflammatory bowel disease, or other comorbidities. Kidney failure and axial DISH were reported in two brothers. He was not taking vitamin D analogues.

**Figure 2:**
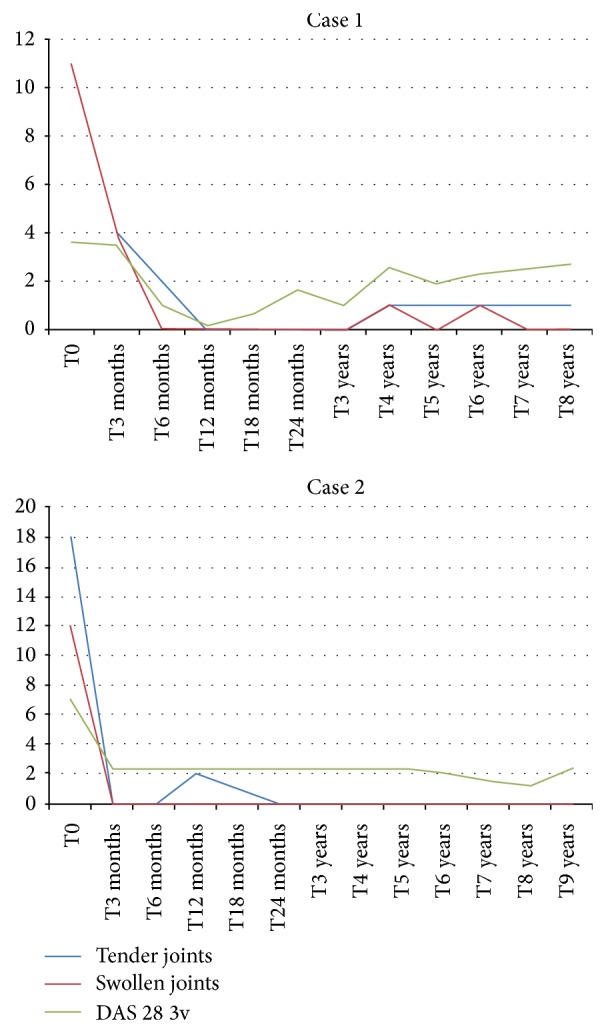
Evolution of the activity indexes in both cases; T0 refers to the first administration of infliximab.

At age 51, he was almost completely in bed with severe arthritis related pain and chronic arthritic disease in the lower limbs, with severe reduction in ankle ROM and flexion deformities of both knees, making walking almost impossible. Symptoms were poorly controlled with analgesics, NSAIDs, and intra-articular and oral steroids. He was therefore started on intravenous infliximab 3 mg/kg each 8 weeks. This resulted in a dramatic improvement with absence of pain and a complete resolution of all tender and swollen joints within 3 months of treatment. Gradual improvement of knee movements led to improved gait and the patient can now walk using a cane and adapted footwear. Over time, discontinuation of all comedications was possible and the patient continues to be treated with infliximab administered every 8 weeks. Significant improvement was observed on the activity indexes until now after nine years of follow-up (Figure 2). ESR and ferritin are now normal. No relevant side effects of infliximab were registered.

### 2.2. Case Report 2

A male patient developed swelling and pain in the MCP joints and proximal interphalangeal joints (PIPs) III, IV, and V of both hands when he was 30 years old, followed by pain in both knees, elbows, and shoulders. Inflammatory lumbar back pain developed around age of 40 years. At age of 50 years, CPPD crystals were identified in a knee effusion and the diagnosis of CPPD chondrocalcinosis was made.

**Figure 3:**
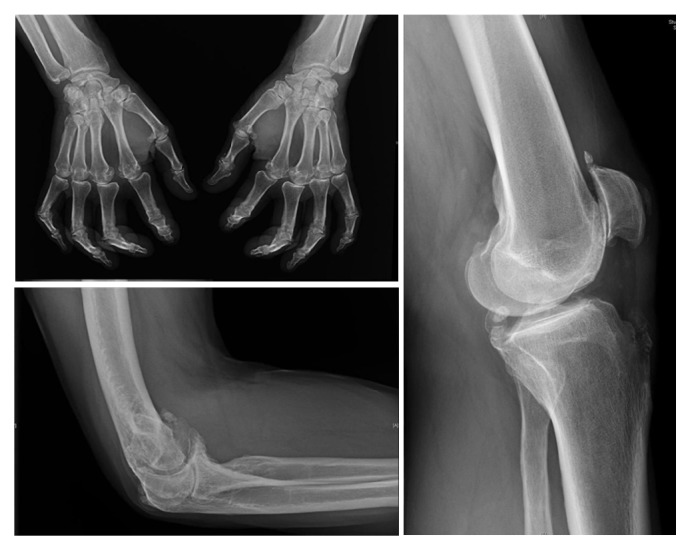
Metacarpal heads osteophytes, MCPs subluxations, and joint narrowing; calcifications of patella and elbow.

By 55 years of age, he had continuous severe polyarthritis affecting fingers, elbows, and knees and flexion contracture of PIPs in both hands with painful forced reduction, important extension limitation of right elbow, and deformity of both tibial spines. X-rays at this stage showed important osteophytosis of anterolateral spine and femoral heads, bilateral patellofemoral arthrosis, subluxations, narrowing, and metacarpal heads osteophytes of MCP II-III-IV in both hands (Figure 3). There was also bilateral periarticular soft tissue calcification of patella, elbow, and inferior calcaneum surface (Figure 3). He had dorsolumbar back pain frequently but no significant restriction of lumbar mobility was found. A similar clinical and radiological phenotype was present in several family members, and pyrophosphate crystals were identified in the knee synovial fluid from the youngest (25 years old) following several episodes of pseudogout.

Laboratory tests showed normal ESR, C-reactive protein (CRP), ferritin, calcium, magnesium, phosphate, PTH, GH, vitamin D, and thyroid hormones (THs). Rheumatoid factor and ANA were negative. He was a H63D heterozygote and HLA-B27 was not identified.* ANKH* sequencing found no pathogenic variants. There was no psoriasis, inflammatory bowel disease, or uveitis. The patient also had a diagnosis of coronary artery disease and atrial fibrillation.

He was refractory to multiple NSAIDs. Intravenous infliximab (3 mg/kg) was started at age 56, each 8 weeks. A major reduction of pain and swelling was observed within weeks, with complete absence of pain in peripheral and axial joints by 4 months. There was a dramatic improvement of hand function with painless reduction of flexion contracture of PIP. There was also improvement in the activity indexes (Figure 2). No side effects of infliximab were observed.

## 3. Discussion

Current understanding of how CPPD crystals interact with the caspase-1-activating NACHT, LRR, and PYD domains containing protein 3 (NALP3) inflammasome of the innate immune system has given novel insights into the mechanisms by which these crystals cause episodic joint inflammation. Nucleotide-binding oligomerization domain- (NOD-) like receptors (NLRs) such as NALP3 have been found to sense endogenous stress signals known as damaged associated molecular patterns or DAMPs [14, 15]. Among several NLRs that form inflammasome platforms, the most studied are NALP1, NALP3 (NLRP3), and IPAF. Pyrophosphate and monosodium urate monohydrate (MSUM) crystals responsible for acute and chronic crystal arthropathies were identified as danger signals activating the caspase-1-activating NALP3 inflammasome, inducing pro-IL-1*β* maturation in macrophages [16].

A role for TNF in crystal induced arthritis has been suggested by the demonstration that exposure of mononuclear and synovial cells to either monosodium urate monohydrate (MSUM) or CPPD crystals leads to TNF-*α* production that is able to stimulate the migration of neutrophils from circulating blood into the joints [17, 18]. Human neutrophils at inflammatory sites are an important source of inflammatory cytokines, and synovial fluid from diseased joints from patients with gout or pseudogout contains large numbers of neutrophils which migrate from circulating blood into the joint. The interaction of CPPD or MSUM crystals with neutrophils at the synovial joint is thought to give rise to the inflammation found in these diseases and to increase the production of TNF-alpha, IL-8, and GM-CSF, which may also increase the migration of neutrophils from the circulating blood [19, 20].

Infliximab is a chimeric monoclonal anti-TNF antibody used to treat several inflammatory diseases, namely, the spondyloarthropathies (ankylosing spondylitis and psoriatic arthritis), rheumatoid arthritis, and inflammatory bowel disease [12, 13, 21]. It is not part of standard care protocols for patients with CPPD chondrocalcinosis. To our knowledge, we are reporting the first two cases of patients with severe CPDD treated with infliximab. Both patients had a peculiar phenotype, already described by several authors, with enthesopathic calcifications, in both peripheral and axial skeletons, which were initially treated with NSAIDs and analgesics without significant improvement. Treatment with infliximab leads to a rapid reduction in pain and improvement of function in both patients, and this improvement was maintained until now (9 years). There were no major side effects and patients now only take analgesics and NSAIDs occasionally in addition to their infliximab treatment. Furthermore, in the first patient, after infliximab treatment, ESR and ferritin returned to normal levels. In both patients, after nine years of follow-up, anti-TNF treatment was shown to be effective and safe.

## 4. Conclusion

We suggest that the efficacy of TNF-inhibitor therapy in severe CPDD arthropathy should be further investigated.
